# Maternal health in rural Pakistan after USAID funding cuts: emerging risks and policy implications

**DOI:** 10.1097/MS9.0000000000004962

**Published:** 2026-04-23

**Authors:** Muddassir Syed Saleem, Adnan Zafar, Muneeb Khawar, Abedin Samadi, Ahmed Asad Raza

**Affiliations:** a Department of Medicine, Karachi Medical and Dental College, Karachi, Pakistan; b Department of Pediatric Pulmonology, John Hopkins Aramco Healthcare, Dhahran, Saudi Arabia; c Department of Medicine, King Edward Medical University, Lahore, Pakistan; d Department of Medicine, Kabul University of Medical Sciences Abu Ali Ibn Sina, Kabul, Afghanistan; e Department of Medicine, Jinnah Sindh Medical University, Karachi, Pakistan

**Keywords:** family planning services, maternal mortality, rural Pakistan, Sustainable Development Goals, USAID funding cuts

## Abstract

Maternal health plays a pivotal role in the achievement of sustainable development, with significant implications for the well-being of societies globally. This editorial explores the critical intersection of maternal health and the United Nations Sustainable Development Goals (SDGs), particularly focusing on the reduction of maternal mortality and the prevention of child deaths. Based on an in-depth review of national reports and the United States Agency for International Development (USAID) data, the role of international aid and the contributions of USAID to maternal health in Pakistan, where funding cuts have led to the closure of essential health facilities and a resurgence of health challenges, has been examined. The editorial discusses the impact of these cuts on maternal health outcomes, especially in rural areas, and highlights the importance of local public–private partnerships in sustaining maternal health programs. Furthermore, it addresses the need for continued investment in family planning services, healthcare worker training, and infrastructure development to ensure the long-term success of maternal health initiatives. The editorial concludes by calling for coordinated efforts to mitigate the adverse effects of foreign aid reductions and to promote sustainable solutions for improving maternal health in Pakistan and other similar settings.

## Introduction

Maternal health’s role in achieving sustainable living has long been the crux of important alliances worldwide. After the drawing up of the 17 Sustainable Development Goals (SDGs) by the United Nations (UN) in 2015 and adopted by all 193 member states, maternal health is now more than ever held on a pedestal as the cornerstone for healthy societies. Goals 3.1 and 3.2, in particular, specifically call for reduced maternal mortality ratio and to end preventable deaths of newborns and children, which are also juxtaposed as a mirror to the general quality of healthcare of nations[1]. According to the World Health Organization (WHO), 95% of maternal mortalities, most of these deaths being from preventable pregnancy and childbirth complications, are attributed to middle- and low-income countries, with sub-Saharan Africa and Southern Asia bearing the brunt of these outcomes according to recent reports^[^1,2^]^. A survey funded by the United States Agency for International Development (USAID) and conducted by the National Institute of Population Studies shows appreciable demographic variations in maternal mortality rates of women residing in rural and urban areas of Pakistan, with the ratio in rural areas being nearly 26% higher. The overall maternal mortality ratio in Pakistan is 186 deaths per 100 000 live births[3]. These statistics highlight the importance of the issue of maternal mortality in the global and local healthcare community in Pakistan, which serves as a driving force behind the inflow of support. SDG 1 – Poverty Eradication – and 4 – Education for All – are also interlinked with the broader objectives of SDG 3, as UN Women highlights that safe motherhood keeps women economically active and protects vulnerable households from healthcare-related financial burden[4]. Improved maternal health outcomes also generate broader intergenerational benefits. Evidence from global analyses shows that maternal education and well-being are strongly associated with improved child survival and development outcomes, highlighting the wider social and developmental benefits of investments in maternal health[5].

## Methodology

This editorial is based on a narrative desk review of publicly available reports, policy documents, and peer-reviewed literature addressing maternal health systems and international aid in Pakistan. Sources published between 2010 and 2025 were identified through searches of PubMed, Google Scholar, and institutional repositories of international organizations, including the World Health Organization (WHO), United Nations Population Fund (UNFPA), and USAID. Priority was given to national demographic surveys, government reports, and peer-reviewed studies examining maternal mortality trends, family planning services, health workforce training, and maternal health program implementation in Pakistan. Relevant policy reports and program evaluations were also included to contextualize the potential implications of recent reductions in foreign aid on maternal health service delivery in rural Pakistan. This analysis draws upon the recent USAID report published in March 2025, which provides comprehensive data and insights on maternal health outcomes and programmatic interventions in Pakistan[6].

## Background

### Foreign aid and its impact on maternal care- *infrastructure*

Much like many other factions of the healthcare system, USAID has been involved in developing maternal health in Pakistan and other lower-income countries. The goals of these programs are to improve maternal and child health and set a foundation for a stable health program for the country to sustain itself in the future. Between 2010 and 2020, Pakistan received approximately $2.5 billion in funding from USAID, with hundreds of millions allocated annually to various sectors, including critical health initiatives[7]. The US government financed the construction of the Jacobabad Institute of Medical Sciences in 2014 and funded the $3.44 million construction of a 60-bed obstetric/gynecological ward and training facility at the Jinnah Postgraduate Medical Center (JPMC) in Karachi in 2012, offering a wide range of services that mothers require. Additionally, USAID funded the construction of another 120-bed maternity ward to replace the 80-year-old maternity ward at JPMC in 2016[7]. These are parables of a fruitful and longstanding agreement of support and mutual dedication to healthy mothers and babies worldwide. However, despite this support, Pakistan remains one of four countries responsible for 47% – nearly half of global maternal deaths according to a WHO-led report. The report further noted that Pakistan accounted for 11 000 maternal deaths in 2023, representing about 4.1%, underscoring the severity of the country’s service delivery gaps that will undoubtedly be exacerbated after removal of aid from a country already struggling to achieve sustainable reductions in maternal mortality[8].

#### Implications

German broadcaster DW and UN agencies warn that maternal health clinics in Peshawar and rural Sindh have closed following USAID cuts, severely reducing service availability and which may significantly weaken maternal health service delivery[9]. Data specific to Pakistan are still largely anecdotal; however, an April 2025 UNFPA report states that maternal, neonatal, and child health services are being severely impacted by humanitarian funding cuts in many parts of the world. Budget cuts have resulted in the closure of medical facilities, the loss of medical personnel, and the disruption of supply chains for medications and life-saving supplies, including those for malaria, pre-eclampsia, and hemorrhage – all of which are major causes of maternal mortality[10]. Reductions in maternal health funding may also threaten postpartum care services, including monitoring for postpartum hemorrhage, sepsis, and hypertension, which are major contributors to maternal mortality in low resource settings.

### Foreign aid and its impact on maternal care– *family planning*

Family planning measures are imperative for the development of any nation facing exceptional growth rates, and the lack of education in rural areas, in particular, has led to the development of poor maternal health outcomes that the country is infamous for[3]. Most recently, USAID awarded Pakistan $40 million via Pathfinder International to reinforce primary health care and encourage the use of voluntary family planning and maternal, newborn, and child health services in Sindh and Khyber Pakhtunkhwa provinces as part of a program that would run till 2027[10]. In the past, USAID provided approximately $60.4 million via the FALAH project to increase demand for and utilization of birth spacing and family planning services across 26 districts in four provinces and reached over 2 million married women and men with family planning information, trained numerous public and private healthcare providers, and expanded the contraceptive method mix available to the population[11].

#### Implications

National family planning service statistics indicate declines in contraceptive service delivery in some public sector programs. For example, recent national reporting shows a reduction of approximately 20% in contraceptive service delivery through the Lady Health Worker (LHW) program, highlighting the fragility of reproductive health outreach systems in Pakistan[12]. Forecast modeling by Cavalcanti *et al*, published in the Lancet estimates that continued USAID aid withdrawals could cause up to 14 million excess deaths worldwide by 2030, including those from maternal and perinatal conditions. Furthermore, reproductive health faces the highest budget cuts of up to 94%. Given Pakistan’s existing high unmet need and fragile family planning infrastructure, any decline in service outreach will correlate with increased unintended pregnancies, riskier births, and maternal mortality[13]. An estimation of the impacts of the 2025 USAID cuts carried out by Sully *et al* reveals that denied access to contraceptive care will see a rise in unintended pregnancies and deaths from complications during pregnancy and childbirth[14]. Based on these estimations, the seizing of the flow of contraceptive machinery and worker training would put the country with already out-of-proportion birth rates and maternal mortality at greater risk than the current Pakistani government infrastructure can compensate for. At this point, extended on-ground realities are difficult to determine especially due to lack of a centralized database and dependency on third-party reports.

### Future landscape

USAID has helped deliver sustainable rural health services and advanced health facility-based childbirth, reducing unsafe home deliveries and directly improving the lives of mothers and children. The disproportionate population growth in Pakistan means that young mothers right now need heavy socioeconomic support to raise a healthy child. Average households require these mothers to return to work early to provide for a young family[15]. Delivery method for these mothers remains a concern and demonstrates a significant disparity that mirrors other limitations in rural areas. According to a health Survey, only 11.5% of rural women had a C-section compared to 25.6% of urban women, and addressing this disparity depends on optimizing the Robson’s criteria to ensure optimal conditions are provided when needed and on foreign support to uphold its guidelines and train local health workers^[^15,16^]^. In total, 57% of Pakistan’s public teaching hospitals have institutionalized the Robson’s Criteria and it has been poised for greater intake. Prior to the initiative, knowledge with regard to Robson’s criteria was scarce and misconceptions regarding C-sections were common. Training and capacity building for increased maternal surveillance will also face scale-downs and cancellations due to new budget cuts, leaving this development area uncertain[16]. Several USAID-supported programs had integrated Robson classification into maternal surveillance efforts, particularly in provincial hospitals. The Robson framework was applied in tertiary-level institutions in Punjab, Sindh, and Khyber Pakhtunkhwa[17]. Candidate recruitment into maternal health training programs, training workshops and tools were introduced to improve monitoring accuracy and curb unnecessary C-sections. Following the suspension of aid many of these structured training modules have been discontinued. USAID-supported programs provided stipends, accommodation, and guaranteed placements for midwifery and nursing graduates, particularly in underserved areas. Since the exit, several provincial institutes have reported declines in enrollment in maternal and neonatal care certification programs following funding cuts. This gap in the training pipeline poses long-term risks to the continuity of skilled birth attendance, especially in high-risk districts. The resulting lack of supervision and reporting has made it increasingly difficult to maintain accountability, particularly in rural areas with high maternal risk, threatening to widen the already stark disparities in surgical obstetric care between urban and rural populations[17].

### Government response

The Pakistani federal government has not yet introduced a national-level contingency plan to replace the maternal health investments previously provided by USAID. Sindh’s Health Department reportedly redirected allocations from its primary healthcare budget to sustain midwife outreach and maternal care activities in rural districts[18]. Still, these efforts have faced substantial operational constraints. These provincial programs suffer from underfunding, irregular disbursement, and limited catchment areas, especially in Balochistan and Khyber Pakhtunkhwa[19]. As of mid-2025, no federal policy framework or national maternal health emergency task force has yet been enacted to address this issue and its potentially serious consequences for maternal health outcomes.

### Conclusion – future recommendations

Health inequality is a concern in developing nations like Pakistan, and although recent improvements point to a trend of reduced disparity in maternal health services in general as well as increased rural inclusion, this improvement is at a significant risk of upheaval with cuts to foreign aid[20]. Leveraging the LHW program may alleviate the disparities faced by marginalized communities as it has been historically documented to improve maternal health outcomes. According to a 2015 evaluation by the Population Council, the presence of LHWs was associated with a 14% higher likelihood of facility-based deliveries and an 18% higher use of modern contraception. The program’s community-based nature allowed it to reach marginalized populations, bridging the gap between communities and the health system. The program’s effectiveness has been in decline during recent years due to resource constraints and political interference[21]; however, the exit of foreign partners such as USAID offers incentive for the Pakistani Government to consider reallocating budgets to this program to help sustain maternal care. The government may consider establishing a national maternal health fund and integrating maternal health into disaster preparedness and broader health policy planning. Local nongovernment organizations (NGOs) such as the Aga Khan Health Services, Indus Health Network, and Rahnuma-Family Planning Association of Pakistan have partially filled gaps by mobilizing mobile health units, family planning services, and community outreach^[^22–24^]^. Additionally, organizations like Health and Nutrition Development Society, Greenstar Social Marketing, and Marie Stopes Society Pakistan have worked to deliver maternal care, midwifery training, and contraceptive access in under-resourced areas^[^25–27^]^. Provincial Governments may collaborate with local NGOs for better penetration into the community, with international partners being on-boarded for not only financial assistance but capacity building and technical assistance as well, possibly as part of larger development deals. Better prenatal and postnatal surveillance optimized with the integration of electronic medical records serve as the cornerstone to a better postpartum period and healthy life for the child.

To ensure methodological transparency and ethical compliance in manuscript preparation, the authors adhered to the recently developed TITAN (Transparency in the Reporting of Artificial Intelligence) 2025 guidelines, which outline standards for responsible AI use in biomedical writing and publishing[28].

## Data Availability

No datasets were generated or analyzed during the current study.
